# Latest Theories of Dental Caries

**Published:** 1891-07

**Authors:** 

**Affiliations:** Pittsburgh


### Latest Theories of Dental Caries.

BY DR. CALHOON, OF PITTSBURGH.

Read before Pittsburgh Odontographic Society.

Before noticing specially the latest theory or theories of dental caries, perhaps in will be interesting as well as instructive, to notice briefly some of the stages through which the etiology of dental caries has passed before reaching the latest and apparently well established theory.

The authors to which we are indebted for the principle data in this paper are Drs. G. V. Black, W. D. Miller and A. Morsman. Also references from brief articles in the dental journals by different authors.

The subject of dental caries seems to have attracted the attention of ancient writers. But the works of Bourdett and Jourdain appearing about the years of 1754–1766 seem to be about the first to treat the subject from a scientific standpoint.

Mr. Fox in 1806 wrote on the subject, Mr. Bell in 1829 and Drs. Fitch and Koecker about the same time. All these writers considered decay of the teeth to be the result of inflammatory action either in the dental pulp, in its membrane or in the substance of the dentine. They supposed it to be of the same character as necrosis of bone. Koecker seems to be the first to acknowledge any chemical agency in the process and that only after inflammatory action had destroyed the life of the part.

About 1830 the inflammatory theory was attacked by a large number of intelligent dentists, the principles ones being Harris, in America; Robertson, in England and Regnard. in France.

In 1835 Robertson, of Birmingham, England, published a work in which he denied the agency of inflammation as a factor in dental caries and asserted that it was a chemical disintegration of the tooth substance caused by an acid genreated by the decomposition of food or fluids of the mouth allowed to collect about the teeth.

In 1838 Regnard, of Paris, published a work taking the same ground as Robertson, more specifically stating that the decomposition of the tooth took place at the spot at which the acid was formed.

Since Schwann, Schroeder, Lister, Koch, Klein and Miller by their careful and extensive experiments and microscopical examinations have demonstrated the agency of micro-organisms in the process of fermentation and putrefaction. It seems to be considered one of the new theories when it is only a fuller explanation of the theory advanced by Robertson and Regnard.

Dr. A. Morsman, in his papers on dental caries published in several numbers of the *Dental Cosmos*, enumerates the following:

1. The vital or inflammatory as the principle theories. 2. The chemical. 3. The chemico-vital. 4. The parasitic. 5. The chemico-parasitic. 6. The chemico-putrefactive.

Dr. W. D. Miller, in his work on micro-organisms of the human mouth, enumerates ten different theories as follows: 1. Depraved juices accumulating in the teeth. 2. Disturbances of nutrition. 3. Inflammation. 4. Worms. 5. Putrefaction. 6. Chemical dissolution. 7. Parasites. 8. Electro-lytic decomposition. 9. Diverse causes. 10. Chemico-parasitic influence.

The latest theory and the one supported by Dr. Black and others and the one which seems to be fully demonstrated by Dr. Miller's experiments is the germ theory.

Many predisposing causes for dental caries are given. The principle ones probably are 1st, imperfect formation of the teeth in which we find deep sulci, pits and grooves making harbors in which the fungi of dental caries collect. Another, irregularities of the teeth, forming spaces which it is almost impossible to keep clean. Uncleanliness may also be mentioned. Others might be mentioned. These are probably the most frequent ones.

But to speak more specifically about the germ theory. By experiments it has been found that there are three kinds or species of micro-organisms or bacteria in decayed dentine. Micrococcus, bacillus and spirillum, the first being the most numerous. Also that there are three separate ferments produced by the fungi of dental caries. 1. Changing a non-fermentable sugar into a fermentable one. 2. An acid producing ferment. 3. A peptonizing or digesting ferments.

It has also been found that the waste product of the above mentioned bacteria is principally lactic acid. In accordance with the above facts, my understanding of the germ theory is that one of the three mentioned bacteria or fungi find a lodgement in some of the harbors, where they are allowed to remain. The first process being the changing of non-fermentable substances to fermentable ones. 2. The formation of lactic acid principally, although other acids are formed, which dissolves the enamel and inorganic substance of the dentine. As the enamel prisms loosen and fall apart the fungi penetrates the enamel until the dentine is reached when its ravages are more rapid on account of there being less inorganic matter. As the bacteria penetrate the tooth they receive their food from without and their waste product being acid the inorganic matter is dissolved before them. 3. The digestive ferment dissolves the organic matter which is washed away or perhaps supplies food for the bacteria.

The different shades of color varying from white to black are accounted for by the progress of decay. The white indicating the most rapid and the black the slowest. The intermediate shades indicating the rapidity of the progress of decay.

Novel Treatment of Ingrown Toenail.—Dr. Puerckhauer recommends a novel, simple and at the same time competent treatment for ingrown toenail. A 40 per cent. solution of potassa is applied warm to the portion of the nail to be removed. After a few seconds the uppermost layer of the nail will be so soft that it can be scraped off with a piece of sharp-edged glass; the next layer is then moistened with the same solution and scraped off; this must be repeated until the remaining portion is as thin as a sheet of paper, when it is seized with a pincette and lifted from the underlying soft parts and severed from the other half. The operation does not require more than half an hour's time, is painless and bloodless, while the patient is delivered from his suffering without being disabled even for an hour.—*The Pittsburg Medical Review.*

